# Attitudes towards human papillomavirus vaccination among African parents in a city in the north of England: a qualitative study

**DOI:** 10.1186/s12978-016-0209-x

**Published:** 2016-08-22

**Authors:** Edith T. Mupandawana, Ruth Cross

**Affiliations:** 1 Yorkshire Mesmac, 22-23 Blayds Yard, Leeds, LS1 4AD UK; 2 Leeds Beckett University, 518 Calverley, Portland Way, LS1 3HE UK

**Keywords:** HPV, Cervical cancer, Childhood vaccination, Culture

## Abstract

**Background:**

Human papillomavirus (HPV) is sexually transmitted and has been conclusively linked to cervical cancer and genital warts. Cervical cancer is attributed to approximately 1100 deaths annually in UK, and is the second most common female cancer globally. It has been suggested that black African women are more predisposed to HPV infection and cervical cancer. A vaccine has been developed to reduce HPV infection, and in the UK, has been offered to 12–13 year old adolescent girls through schools as part of their childhood immunization programme since 2008. Upon programme initiation, it was noted that vaccine uptake was lower in schools where girls from ethnic minority groups were proportionately higher.

The study’s objectives were to explore factors influencing UK based African parents’ acceptance or decline of the HPV vaccine, whether fathers and mothers share similar views pertaining to vaccination and any interfamily tensions resulting from differing views.

**Methods:**

A qualitative study was conducted with five African couples residing in north England. Face to face semi-structured interviews were carried out. Participants were parents to at least one daughter aged between 8 and 14 years. Recruitment was done through purposive sampling using snowballing.

**Results:**

HPV and cervical cancer awareness was generally low, with awareness lower in fathers. HPV vaccination was generally unacceptable among the participants, with fear of promiscuity, infertility and concerns that it’s still a new vaccine with yet unknown side effects cited as reasons for vaccine decline. There was HPV risk denial as religion and good cultural upbringing seemed to result in low risk perceptions, with HPV and cervical cancer generally perceived as a white person’s disease. Religious values and cultural norms influenced vaccine decision-making, with fathers acting as the ultimate decision makers. Current information about why the vaccine is necessary was generally misunderstood.

**Conclusion:**

Tailored information addressing religious and cultural concerns may improve vaccine acceptability in African parents.

## Background

The humanpapilloma virus (HPV) is sexually transmitted and has been conclusively linked to cervical cancer (CC) and genital warts [1, 2]. Multi-parity, early sexual debut and infection with other sexually transmitted infections (STIs) are known to be risk factors for HPV and CC [3]. HPV infection is so common that it’s suggested that 20 % of sexually active adolescent girls will be infected by the age of 18 [4]. As 70 % of infections clear within a year however, only a small proportion of women who develop persistent infection from high risk genotypes go on to develop cancer [5]. CC is attributed to approximately 1100 deaths annually in the United Kingdom (UK) [6]. CC ranks second in the most common female cancers globally [7], and is the main cause of female cancer mortality worldwide [8], especially in Sub-Saharan Africa [9].

Since 1979, CC mortality rates in the UK have reduced by nearly 70 % due to the introduction of the cervical screening programme in 1988; and more recently the HPV vaccination programme [10]. The HPV vaccine has been proven to reduce the risk of CC [11]. Nearly all the girls vaccinated against the HPV virus will be protected against three-quarters (75 %) of CC incidences [10]. To increase efficacy, the vaccine must be given before sexual debut [12]. It has been suggested that adolescents are becoming sexually active earlier [5], therefore, in the UK, the HPV vaccine has been offered to 12-13year old girls since September 2008 through schools, as part of their childhood immunization programme [13]. HPV vaccination programmes together with cervical screening are expected to reduce CC incidence rates and mortality rates [14], thereby reducing disease burden on the victims of CC and their families [15]. It is noted that programme success is influenced by awareness and that lack of awareness impacts negatively on uptake [16]. Raising awareness in parents is central to influencing HPV vaccine uptake as they are the decision makers [17]. Lack of parental awareness can result in vaccine decline, and school nurses, who are the primary deliverers of the vaccine, have expressed reluctance to vaccinate adolescent girls without parental consent [5]. Consequently, parents of adolescent girls need to be aware of HPV, and how it is transmitted and the efficacy of the HPV vaccine in preventing CC [17].

Generally, awareness in the UK has increased since the introduction of the school based vaccination programme which was preceded by various health campaigns [18]. A study by Marlow et al. [19], reported a positive correlation between having a daughter aged between 9 and 17 years and increased awareness in UK parents. Women are reported as having higher levels of awareness of both HPV and the vaccine than men [19], however, black women are reported to have lower levels of HPV awareness compared to their white counterparts, and less likely to have heard about the HPV vaccine [18]. Previous studies have indicated that black women from Sub-Saharan Africa have a limited knowledge pertaining to HPV and CC [9, 17]. It is argued that limited awareness will invariably affect vaccine acceptability and therefore vaccine uptake [18] and that poorer knowledge pertaining to CC, and its link with sexual activity and HPV is associated with vaccine refusal [13].

Although there is a generally good uptake of the vaccine, a feasibility study conducted prior to the nationwide initiation of the programme, indicated significantly lower vaccine uptake in schools where students from ethnic minority groups were proportionately higher [20]. More specifically, Hawker et al. [21] suggested that black parents have poorer uptake of childhood immunizations, falling behind their Asian and white counterparts. This is a cause for concern. It is argued that CC is a disease of disparities, with factors such as race, ethnicity, socio-demographic and socioeconomic factors playing an important epidemiological role [16] with suggestions made that women from ethnic minority groups are particularly vulnerable to CC [3, 15].

Such findings suggest that research into the attitudes of minority groups towards the HPV vaccine is necessary. Gordon et al. [13] carried out research with the Jewish population in the UK, but no UK studies appear to have been carried out with the African population. Most research in this field has been based in America. Internationally, several studies have been conducted exploring the attitudes of mothers towards HPV vaccination, but fathers have generally been neglected. A study by Marlow et al. [1] found that mothers who thought their partners would be favourable to vaccination were likely to accept vaccination for their daughters. The male presence within the family unit is typically one of authority, thus a father holds the greater power in the decision-making process in all matters including sexuality, reproduction and matters pertaining to the children [22]. This is of particular importance in the African context, where the man in any relationship is the decision maker [22], making it impossible for mothers to act without permission from their husbands [23]. Generally, African women hold a lower social status and are disempowered, and the African tradition favours this [23]. This brings to the fore the importance of fathers in the decision making process in relation to HPV vaccination.

In addition to issues of gendered power Wamai et al. [17] suggest that other social and cultural factors influence HPV, HPV vaccine and CC awareness. Attitudes and beliefs similarly influence vaccine acceptability [24]. There has been a resurgence of religion within many Africans in previous several decades, possibly born from a desire by Africans to search for salvation/escape from their impoverished socioeconomic conditions [25]. Religious beliefs dictate no sex outside of marriage, and these beliefs can impact on risk perception, and ultimately vaccine acceptability [13]. Religious values may make it difficult for parents to find acceptable any interventions that seem to be linked to sexual behaviour [25]. A study by Gordon et al. [13], found that religious beliefs played a pivotal role in the decision-making process in matters pertaining to sexual behaviour.

In addition, compared to other races, black women are reported as being less receptive of the HPV vaccine [18], amid concerns about possible side effects and uncertainty of the vaccine’s effectiveness [26].

For the reasons outlined, the present study sought to explore attitudes towards HPV vaccination among UK based African parents of daughters aged between 8 years and 14 years.

The study had three objectives:

- To explore whether African parents in the UK have an awareness of what HPV vaccine is, and how the virus is transmitted and also to identify their sources of information. This was important so as to explore whether lack of knowledge and awareness impact on the decision making process; knowledge will likely influence uptake [24].
- To explore the attitudes towards and acceptability of HPV vaccination by UK based African parents, and the factors influencing their acceptance of the vaccine including whether cultural principles influenced decision making, taking into account that in the African context, culture plays an important role in health behaviour [27].
- To explore whether mothers and fathers have similar views about their daughters having HPV vaccination, and any inter-family tensions around consenting to HPV vaccination.

## Methods

Participants were recruited and interviewed by ETM. The study adopted a descriptive qualitative design. Face to face semi-structured interviews were conducted allowing for the collection of rich, in-depth data [28]. Purposive snowball sampling was used to select the participants who were recruited via gatekeepers from an African social club in a city in the north of England. Given the focus of the study, participants had to be African parents with at least one daughter aged 8–14 years. Single parents where the other parent was not contactable for research purposes were excluded as it would not have been possible to address the third objective of the study. Divorced, separated, and families where one parent was not willing or unable to participate in the study were also excluded for the same reason, and were not recruited. Willing participants were provided information sheets outlining the study; that participation was voluntary, that their anonymity would be maintained (pseudonyms were used); when and how they could withdraw from the study; that interviews would be tape-recorded and how and when they would be destroyed, and were given 5 days to consider whether or not they wished to participate. No incentives were offered for participation.

Interviews were conducted in study rooms located within the university library. The interview duration was approximately one hour long. The interview schedule was based on the study objectives, and covered topics such as how long they have lived in the UK, number of daughters, their understanding of cervical cancer, their understanding of HPV and the vaccine, what they think is the appropriate age for vaccination, and their spouse’s perceptions about vaccination, and their ability to discuss vaccination with their spouse. Discussion topics also included who makes decisions within the family, and if they have any daughters already vaccinated, and reasons for any previous vaccine refusal. Each participant was interviewed without their partner. After a total number of 5 mothers and 5 fathers were recruited and interviewed, a point of data saturation was reached, i.e. no new information was forthcoming [29]. All interviews were recorded, transcribed, and analysed using thematic data analysis by hand. An inductive approach to the analysis was used. To enhance validity, participants were provided copies of their transcripts to check for accuracy [28]. The sample size was small, and was a limitation of the study.

The male participants were unwilling to provide their exact ages, something which could be resultant from the fact that the interviewer was an African woman. Given the reluctance of the male participants to provide their exact ages on account of the interviewer’s gender, it cannot be entirely ruled out that they may have responded differently to being interviewed by a man. It is unlikely however that their perceptions would have been different had the interviewer been male. The study did not note other demographic factors. It is acknowledged that literature suggests factors such as socio-economic status and educational levels may influence health behaviour [16]. However, in the present study, there were no obvious differences between the attitudes of the participants according to incidental demographic variances which emerged during the data analysis. For example, the attitudes of the nurse from Kenya, despite having a higher level of educational attainment, were in accord with the rest of the cohort.

## Results and discussion

The key findings of the study are now discussed with reference to existing knowledge and previous research findings. A number of themes were identified in the data. The first theme is about factors influencing vaccine acceptability namely, awareness and protection. The second theme is about factors influencing vaccine decline and include links to promiscuity, vaccination age, the newness of the vaccine, conspiracy theories and religion. The third theme is about decision-making processes and power. The final theme relates to perceptions of risk and includes the subthemes risk neutralisation; culture as cervical cancer risk and culture as protection against HPV infection. Each theme is presented and discussed in turn.

### Theme 1: Factors influencing vaccine acceptability

#### Awareness

Vaccination uptake and completion of all doses are influenced to a greater extent by the HPV knowledge and awareness that parents and care givers have [30]. Low et al. [10] suggest that although HPV and CC awareness within UK women is generally low, it is ethnicity associated, with white women having higher awareness. The results of the present study suggested that the majority of participants found the vaccine unacceptable, and demonstrated low levels of HPV and CC awareness. Awareness was higher in the women in this study than the men.

Kontos et al. [30] suggest that parents are relying more on information gathered from the internet, and that in America, African American parents are least likely to search for information on the internet. Interestingly, the present study indicated that the majority of parents received information from schools, with no parents making any reference to the internet as sources of information. The mother from the couple who consented to vaccination reported to having discussed HPV vaccination with her doctor apart from the information received from the school, and they both seemed to have high awareness levels; as she imparted the acquired knowledge to her husband. However, they did mention the internet when they mentioned that they were concerned that their daughters may look up HPV on the internet and thus learn what it was and develop loose morals; this interestingly was the only time the internet was mentioned by any participant;

*“These children nowadays read things on the internet, so if she finds out what the vaccine really does, she might see it as a green card to have sex”* (Patrick, Nigerian father, aged 42 years).

All the parents reported having received information from the school, so they did have some HPV knowledge, although there wasn’t much awareness pertaining to CC. However, even though they had received the information, it was as though there wasn’t much understanding of the information, as most of the parents continued to believe that only promiscuous and sexually active girls should be vaccinated; since lack of sexual activity was cited by a few of the parents as reasons for declining the vaccine. This is contrary to the fact that the vaccine works best before sexual debut [15]. Perhaps the information is inadequate, and if more tailored, uptake may be increased. Similarly, awareness is enhanced by having personal experience or knowing a close friend or relative who had experienced abnormal screening results or cervical cancer [10]. None of the participants in the present study indicated having any personal experience or association with cervical cancer, in fact a few of the participants indicated they did not have such conditions within their lineage and it was dismissed by some as a condition for old women;

*“Cervical cancer at home is seen in old women”* (Marita, Zimbabwean mother, aged 40 years).

*“No one in my family has ever had that and I have never heard of anyone getting it”* (Lucas, Zimbabwean father, mid 40s).

Parents were generally uncomfortable about discussing a sexual health issue with their daughters, and would therefore, not discuss HPV vaccination with their daughters because of its link to sex. The exploration into the reasons why parents were reluctant to get their children vaccinated showed the deep divisions that exist between cultural influence and the acceptance of western culture. Despite several years of residency in the UK which ranged between 7 and 17 years, it appeared that most of the participants held onto their African heritage, and had not embraced the way of life in the UK, and expected their children to do the same. Perhaps this was because the participants were recruited via an African social club. A study by McRee et al. [31] found that American mothers (a western culture), were willing to discuss HPV and sex with their daughters. On the other hand, some of the parents in the present study pointed out that traditionally, other members of the extended family such as aunties were responsible for discussing sexual topics with children, however, migration had eroded this, and parents found themselves having to carry out this role, and the majority found it impossible. The majority of parents expressed that this role was now taken over by the mothers, although most of the mothers found it a challenging task. The African culture looks at sex as a taboo subject and therefore there is limited discussion between parents and children [32] unlike the openness that exists amongst the western population [31]. Consequently some of the parents stated they would simply refer to the vaccination as ‘the injection’, and stated that they would avoid mentioning what the vaccine was for. Whereas the western population is reported as willing to discuss HPV vaccination with their daughters and make a joint decision [33], the present study found that neither of the parents would discuss the vaccine with their daughters, and decisions would be made on their behalf, with the children doing as they are told. These cultural and religious principles also made discussions about the vaccine and associated sexuality difficult between couples. However, a small minority felt they could discuss the importance of good moral behaviour with their children as a way of safeguarding them against HIV and other STIs.

It is not evident whether length of stay in the UK influenced vaccine acceptability. The Nigerian couple had stayed in the UK for 15 and 17 years and accepted vaccination, but the Kenyan couple had been resident in the UK for 14 years, and refused vaccination. It should be noted, however, that the only couple to perceive vaccination as acceptable, had been resident in the UK the longest.

#### Protection

Protection against HPV infection and/or CC offered by the vaccine influenced vaccine acceptability, even by those who did not consent to vaccination. Some of the parents; thought that the vaccine offered necessary protection to their children, and one Nigerian couple would have wanted their son to be vaccinated as well. Some mothers thought the vaccine was necessary, but felt powerless to consent to vaccination due to the fact that they felt they could not override their husband’s wishes;

*“I think the vaccine will help her not get cancer and live long. If it was up to me I would consent, but I am not the head of the family. Her father said no”* (Marita, Zimbabwean, aged 40 years).

The fact that a well-behaved child may become infected by the man she marries influenced vaccine acceptability and acceptance for the Nigerian couple;

*“…she can be infected by the one boy she sleeps with or the man she marry. So because my daughters are precious gifts from God, I don’t want to take any chances”* (Mary, Nigerian mother, aged 38 years).

This supports findings from Galagan et al. [34] who demonstrated that knowledge of the protection offered by the vaccine influences acceptance of it. This is a typical example of the Protection Motivation Theory (PMT), where the parents believed that their children were at risk of acquiring the infection, and that the vaccine offered real protection [35]. Even among the religious British-Jewish community, some mothers felt it best to protect their daughters against HPV infection since they could not guarantee that they would lead a Jewish lifestyle [13]. However, as noted in this study, risk perception does not always result in health-seeking behaviour due to other constraints, such as gender inequality affecting action by mothers, as well as such factors as religious principles and values.

### Theme 2: factors influencing vaccine decline

#### Link to promiscuity

The majority of the parents interviewed expressed that only those who are promiscuous or intend to be would benefit from the vaccine. It has been suggested that the link between CC and HPV, a sexually transmitted virus has evoked feelings of shame, anger and anxiety; as it has been associated with promiscuity [36]. In the present study however, this link with promiscuity made the vaccine less appealing for most of the participants, with those accepting vaccination still pointing it out as a cause for concern. All participants expressed the concern that vaccination may result in their daughters sleeping around because of the protection offered by the vaccine as they made a clear connection between morality and HPV vaccination;

*“People must know that if they do this thing [consent to vaccination], they are giving their daughters a free licence to sleep around”* (Doreen, Zambian mother, aged 39 years).

This link between vaccination and promiscuity is consistent with a Ghanaian study by Coleman et al. [37]. One Zimbabwean father when asked about any problems he envisaged developing as a result of his daughter getting vaccinated said;

*“Of course! A vaccine for prostitution? She will become a whore, everyone’s horse”* (Lucas, mid 40s).

Even those that consented to vaccination expressed similar concerns, although the latter felt that protection outweighed the risk of immoral sexual behaviour;

*“…it might make her feel that it’s ok to have sex, but…saving her life was more important”* (Mary, Nigerian mother, aged 38 years).

It was also feared that the vaccine may give a false sense of security, risking infection from other conditions like the human immunodeficiency virus (HIV). A study by Martin et al. [12] also indicated similar sexual complacency concerns. In the present study, this fear resulted in some refusing the vaccine, and deciding to focus more on talking to their children about the dangers posed by risky sexual behaviour;

*“I told her if she did not sleep around then she would be safe”* (Ruth, Kenyan mother, aged 44).

It was also anticipated that the knowledge and fear of acquiring HIV would help children maintain good moral behaviour;

*“…and they know about the risks of HIV, so they are not likely to put themselves at risk, I don’t think”* (Ruth, Kenyan mother, aged 44).

On the other hand, this fear of other infections resulted in those consenting to vaccination stating they would consent if there was a vaccine to protect against HIV;

*“…if there was a vaccine to stop her getting HIV, I will sign the paper”* (Patrick, Nigerian father aged 42).

Interestingly, one father viewed HPV and CC as deserved punishment for immoral behaviour;

*“If people want to be promiscuous, let them pay for their sins. Others have paid by dying from HIV, so what’s different here?”*

#### Vaccination age

The majority of participants thought the vaccination age was too young, with most expressing older girls or young women who are sexually active should be vaccinated;

*“I think young women getting married are the ones who should be vaccinated”* (Doreen, Zambian mother aged 39 years).

This was echoed by other parents, although the Zimbabwean father thought that the vaccine would have been better suited to the old generation since they are the ones who were at risk because of multi-parity and traditional medicines. Others, however, thought that the vaccine was being offered at the right age, with one mother stating that the vaccination should be offered earlier since children seem to be sexually active much earlier;

*“I hear girls as young as 11 are having sex with their boyfriends, and you know about those Asian men who were having sex with very young girls, so I think it should be from 10 years to be on the safe side”* (Mary, Nigerian, 38 years).

The issue relating to vaccination age is echoed in other studies [1]. For vaccine efficacy, it has to be administered before sexual debut [12, 15]; it would appear from most of the responses around this that this was not understood by most of the parents, and is an area that needs further study.

#### New vaccine

A majority of the parents felt that the vaccine was still very new and that some of the side-effects of the vaccine were yet unknown; resulting in mistrust for the vaccine. The recent link between measles, mumps rubella vaccine(MMR) and autism, which resulted in vaccine decline by some of the British population was mentioned by a few participants as they felt this vaccine was too new, and one mother stated that it may take as long as 20 years for side effects to manifest;

*“Let them vaccinate their own children first, then after 20 years if nothing happens, we will also vaccinate our own”* (Susan, South African mother, aged 42 years).

Although literature does not suggest any future side-effects to the vaccine, it however suggests that any effects on incidence of the vaccine will only be evident when the vaccinated girls reach the ages of 30–39 when CC is most common, which would be about 18 to 27 years from vaccination [10]. A few of the parents expressed concern over future infertility, which in the African context is a cause for great concern as it can result in marriage breakdown and ostracism [38], and impacted on vaccine acceptability in a study of Ghanaian women by Coleman et al. [37]. In previous studies, the notion that the vaccine is still new also influenced vaccine decline as some mothers feared the unknown side effects to the drug [13]. One mother reported that the death of a school girl who had just received the vaccine influenced vaccine refusal. The same reason has been cited in other studies, although it was suggested that the girl’s death was not related to the HPV vaccine [39]. This highlights the fears a new vaccine may present, as all side effects may not be known; and any consequence whether associated to the vaccine or not, is invariably attributed to it.

#### Conspiracy theories

Various conspiracy theories have impacted on health decision making for some Africans. For instance, the conspiracy theory that HIV was created in an American laboratory targeted at reducing the African population and was delivered to Africans through the polio vaccine affected uptake of polio and other vaccines, especially when they were donated from the west [40]. Similarly, rumours that donated condoms were tainted with HIV affected condom use [22]. These and other conspiracy theories have been reported as influencing decision-making for some parents;

*“Remember this is a white man’s vaccine. The white man brought us AIDS to kill us off because we were too many; now, they might want to make our daughters sterile”* (Susan, South African mother, aged 42 years).

There was an element of scapegoating noted in the data as the white race was blamed for the acquired immune deficiency syndrome (AIDS) and conspiring against the African population [41], and was another form of risk denial. A Kenyan mother, also a nurse reported that when a meningitis vaccine was donated and offered to Kenyan children in 2002–2003, rumours circulated that this was targeted at reducing the population in the next 20 years when the children became of childbearing age, by rendering them infertile. This, she said greatly influenced her decision to decline the vaccine, although she had already left the country at the time the rumours circulated. This shows that people maintain close contact with their countries of origin, and that rumours circulating within countries of origin have far reaching consequences.

The fact that the vaccine was still relatively new influenced vaccine decline; a finding which has been reported in other studies [2]. There was general mistrust of the vaccine, and the western world from which the vaccine was said to originate. Previous vaccination rumours and scandals such as the MMR also influenced vaccine decline.

#### Religion

Religious values greatly influenced the decision-making process, with religion being cited by half the participants as reasons for declining vaccination. Religious beliefs and principles were thought to shape a child’s behaviour such that their risk was greatly reduced due to good moral behaviour;

*“…and all my children are growing up in the church; in the Christian way. The school said it’s [HPV] caused by sex, so my children won’t have sex until they’re married”* (Susan, South African, age 42 years).

Some of the fathers seemed to hold this view too. When asked if he thought his daughters were at risk of acquiring HPV, Moses a South African father in his late 40s said;

*“My wife takes the children to church and they are being raised well, so no”.*

However, in one interesting case, a Zambian mother who made a unilateral decision to decline vaccination did so because she felt unable to discuss vaccination for what she perceived as a sexual thing with her husband;

*“How can I discuss such a shameful thing with baba?”* (Baba meaning father).

The fact that the husband was highly placed within the church hierarchy seemed to make the subject of HPV vaccination more unapproachable. Literature suggests that religious values have affected Africans’ risk perceptions and the acceptability of some discussions, and consequently, in past decades this has resulted in increased HIV/AIDS mortality within Sub-Sahara Africa, prompting calls upon church leaders to speak out for the sake of future generations [25].

### Theme 3: the decision-making process and power dynamics

Different factors contributed to the decision to either accept or decline the vaccine. A few of the participants reported that other parents’ perceptions influenced their decision-making processes, and it was mainly negative influence.

*“…talking to other mums, noone wants their 12 year old vaccinated…”* (Ruth, 44 year old Kenyan mother).

This may have been born from the fact that the mothers they would normally discuss vaccination with would be from similar backgrounds, and thus share similar views. This peer influence is reported in other studies [42].

Notwithstanding, the present study indicated that the decision-making seemed to be centred on who holds the power within the family unit, and in most cases it was the father, rather than the mother, who made the final decision:

*“All decisions are mine; everybody who lives under my roof does what I say”* (Daniel, Zambian father, late 40s);

*“…I’m not the head of the family”* (Marita, Zimbabwean mother, 40 years).

It was evident from most of the participants that the fathers were the ultimate decision makers in most issues pertaining to the family and especially the children. Similar masculine power dynamics are found in other studies [23]. The parents in this study conform to the African gender culture guidelines which stipulate that males should demonstrate masculinity and strength by exerting control over their families [32]. Participants gave the impression that mothers did as they were told, as demonstrated by Daniel, a Zambian father in his late 40s;

*“She thinks what I think- I am the head of the family”.*

Both the fathers and mothers seemed to accept this as the natural state of affairs and fostered a general acceptance that both the children and the wives belong to the father [23]. Although the men were the decision-makers in all other matters, they seemed to relegate knowledge pertaining to early childhood vaccinations to the mothers as they felt it was part of their care-taking role. This seemed contradictory to their standing as the ultimate decision-makers, and the claim that their children belonged to them. Similarly, some of the fathers were not sure about their daughter’s ages, which was interesting since both they and the mothers portrayed that the children belonged more to the fathers than they did the mothers. The results from this study in relation to the power dynamics in decision-making and influence to consenting to vaccination, contrast with a South African study by Francis et al. [9], which found that mothers did not mention lack of paternal consent as a reason for not vaccinating, but rather that health care providers are the ones who played a pivotal role. Furthermore, a different study by Francis et al. [43] indicated that mothers and grandmothers were the decision-makers in matters pertaining to health, whilst the fathers played a minimal role or did not partake in the process. These findings from both studies by Francis et al. contradict with what literature suggests as the norm within the African context [23, 44].

Although fathers were happy to relegate the responsibility for early childhood vaccination to mothers, in the case of HPV vaccination, they seemed to reclaim the parental responsibility, perhaps because of its link to sex. One father from Zambia mentioned that he didn’t know anything about vaccinations, as they were the mother’s responsibility, however vaccinating his daughter against HPV was his business;

*“I don’t know anything about vaccinations. It’s the mother’s responsibility…but injecting my child with anything like this is my business”* Daniel, a Zambian father in his late 40s*.*

Without the authority to decide whether to vaccinate, the majority of mothers expressed that tensions would arise between them and their husbands if they went against their decisions to decline vaccination; even those that felt that vaccinations were necessary for their daughters. Some even feared being divorced by their husbands as a result.

*“If I consented against his wish and something happened to her, or he found out about it, he would divorce me. Accuse me of being promiscuous and rebellious”;* (Marita, 40 year old Zimbabwean mother).

Interestingly, this fear of rejection extended beyond her husband and included her extended family;

*“…my family would be ashamed of me and maybe reject me”;* (Marita, 40 year old Zimbabwean mother).

Even where vaccination was accepted, there was still the notion that the father was the ultimate decision-maker;

*“My husband is an African man, so he must be involved in any decision especially when it concerns his children. …in my own culture, they are more his children than they are mine”* (Mary, Nigerian mother, aged 38).

Most of the fathers on the other hand, did not think any tensions would arise, as they expected their wives to do as they were told, and in the event that this was not so, some expressed that they would send either mother or daughter, or both away:

*“If she goes against my word and has her vaccinated, then she should take her to her real father. It will mean that I am not her real father”* (Lucas, a Zimbabwean father in his mid 40s).

Culture has generally placed women under their husband’s authority, and this has influenced how they relate to their husbands and other male relations [23]. This aspect of the African culture which disempowers women makes it difficult for women to seek their own medical attention without approval from husbands or other male relatives, especially as they are usually economically dependent upon the males in their lives [44]. Even where no cost is effected, it is deemed disrespectful for a woman to make any decision by herself without seeking consent from her husband, father or other male relative [23].

The results of this study demonstrate who holds the power in HPV vaccination decision making, and the underlying power dynamics within the African family unit. They also illustrate how both culture and religion play a pivotal role in influencing HPV and CC risk perceptions and vaccine acceptability. The results also indicate mistrust for the relatively new vaccine.

### Theme 4: risk perceptions

Perceptions of personal risk are usually borne from local history, personal experience, social circumstances and lay knowledge within a given cultural context, and influence health decision-making [23]. There was a general lack of HPV risk reported by most of the participants, with religion and good cultural upbringing cited as reasons for upright moral behaviour and therefore reduced risk. Even where there were perceptions of risk, this was not enough to result in vaccination acceptance, as other potential risks such as infertility, death and unknown side effects seemed to outweigh infection risk. This brought to the fore the assumptions within the Health Belief Model (HBM), and Protection Motivation Theory (PMT), where parents had to be aware that their daughters were at risk of HPV infection; that the vaccine would greatly reduce this risk; then consciously weigh the “the pros and cons of taking action and make a decision accordingly” ([45], p.103). Perceived risk is central to action towards prevention in most decision-oriented health behaviour theories [46]. In the present study, only one couple perceived the risk presented to their daughter and decided to act towards protection, however, the majority did not perceive any risk, and where risk was perceived, some parents weighed risk through infection which they may or may not acquire, versus a risk that seemed to be more worrying for them such as future infertility, or other adverse effects they did not know about. A Kenyan mother aged 44 commented that:

*“It’s a new vaccine, nobody knows what the long term side effects are.”*

#### Risk neutralization

In the instances where there was perceived risk of both CC and HPV infection, some of the parents seemed to neutralize these risks in a way that was rational for them. Synonymous with the neutralization theory, some parents denied their daughters’ HPV and CC risks by distancing them from what they perceived as HPV risks [41]. Where traditional practices like multi-parity and medicines were seen as predisposing to CC, parents said their children’s risks were greatly reduced because they would not use these traditional medicines, but would instead use things like soap which were perceived as safer for preparing the birth canal, and also bearing fewer children thus reducing their risk;

*“…to prepare the birth-canal we don’t use traditional medicines anymore, but soap—which is safe”* (Susan, South African, 42 years old).

*“…these days people don’t have many children”* (Daniel, Zambian, aged late 40s).

#### Culture as cervical cancer risk

Some of the participants attributed CC risk to African cultural practices and not HPV. Cultural practices such as using traditional medicines in the vagina for various reasons like tightening the vagina to enhance sexual pleasure for their male partners, and preparing the birth passage were some of the CC risk factors cited by the participants;

*“Its [cervical cancer] caused by the medicines women wear [insert in their vaginas]. The medicines to tighten yourself so that your husband can enjoy you more, and also to prepare passage for childbirth, and then tighten again after delivery”* (Doreen, Zambian, 39 years).

This point was echoed by other mothers. The belief that the use of traditional medicines is a cause for cervical cancer has been reported in other studies [36]. The culture of bearing many children to help with the field work, and also to protect marriages was reported by a few participants as a cause for CC;

*“Long ago women had plenty children. If they didn’t have a lot of children, [then] who will work in the fields?”* (Daniel, Zambian father, aged late 40s).

*“To protect her marriage a woman can keep having children until a son comes, otherwise her husband can get the son from somewhere else”* (Susan, South African mother, aged 42 years).

Consequently most of the participants perceived increased cervical cancer risk in elderly women, as they attributed increased risk to prolonged use of traditional medicines. When asked what she understood about CC and its cause, a Zambian mother stated she had heard that CC kills old women;

*“Old women because it takes a long time for the poisons in the medicines to affect the womb, and they used a lot of those medicines in the old generation because they had more children”* (Doreen, Zambian mother, aged 39 years).

#### Culture as protection against HPV infection

Conversely culture was also cited as protection against HPV infection. A good upbringing was perceived as protection by both mothers and fathers, with HPV infection perceived as an infection for children who come from uncultured, non-African backgrounds. In making a statement that his daughter is not at risk of HPV infection, one father stated;

*“An African child from a good African home? Tell me—have you ever heard of an African child having this disease”* (Lucas, mid 40s, Zimbabwean).

Non-African (white) girls were stereotyped and stigmatized as uncultured and immoral. The fact that the one cervical cancer case that made the headlines was that of Jade Goody, a white British celebrity, seemed to make a few of the parents think this is a white people’s disease;

*“He said the vaccine is for white people’s children who have loose morals, …. He said black girls never get this promiscuity disease”* (Marita, aged 40 years).

*“…children from different ethnic backgrounds behave differently. The white girls tend to be sexually active early, whilst African and Asian girls tend to do so later”* (Ruth, Kenyan mother).

Apart from a good cultural upbringing, ancestors were also cited as offering protection against HPV. This belief in ancestral protection demonstrated external locus of control [47], as fate factors such as ancestral protection are perceived as having a great influence over their and their children’s lives [48];

*“....Besides, our ancestors protect us. The fact that we are in this white man’s land does not mean we loose our roots or that our ancestors forsake us”* (Lucas, mid 40s, Zimbabwean).

## Conclusion

The results of the study indicate that the HPV vaccine is generally unacceptable within the African population, with culture and religion influencing risk perceptions and playing important roles in vaccination decision making. Mistrust for the vaccine was also evident, and a general mistrust for the west from which the vaccine originates. HPV and CC knowledge was low, and there was a misconception pertaining to the causes of CC; all which contributed to vaccine decline. To increase uptake, parents should be provided tailored HPV information that addresses religious and cultural issues specific to the African population [42]. This is important because HPV information from the schools was generally misinterpreted, as some parents continued to believe that it should be administered to girls of questionable moral behaviour only, implying that it works to prevent HPV in those who are already sexually active, contrary to literature. It would appear that the information received from the schools was not understood in its entirety. Africans are people with deep cultural roots, which influence their health behaviour [27], and this together with their religious values should be reflected in health messages. Health information should stress the fact that the vaccine is most effective when given to girls who are not yet sexually active, and importantly that girls can be at risk because of exposure to the virus, not through their own sexual behaviour, but their partners, namely husbands. Mentioning husbands could influence vaccine acceptability, as this does not imply promiscuity on the girls’ part. The interview did not change the perceptions of any of the participants towards HPV vaccination.

Audience segmentation and targeting health communication to smaller more homogenous subgroups can make it easier to determine the factors shared by members of a selected subgroup with increased disease burden, and thus tailor information to suit [49]. Health information with short story lines focusing on religion and HPV vaccination may also improve HPV health literacy, where the target group are able to identify with the characters portrayed in the short skit; identifying with the source of the message, or persons portrayed in the message has been proven to enhance effective communication, and consequently service uptake [49]. Involving key influential people e.g. church leaders from within the targeted community in health campaigns may influence vaccination uptake [34]. Community representatives should be involved in designing a message that serves the needs of the study population [7]. The use of celebrities in health messages may also be beneficial, especially male celebrities, as this might influence vaccine acceptability for fathers, who in the African context are the ultimate decision makers. Furthermore, considering the importance of African fathers in HPV vaccination decision making, they should not be excluded from any future research in this population. As with other qualitative research, the results of this study cannot be generalised and is a limitation of the study.

## Abbreviations

AIDS: Acquired immune deficiency syndrome
CC: Cervical cancer
HBM: Health Belief Model
HIV: Human immunodeficiency virus
HPV: Human papillomavirus
MMR: Measles, Mumps and Rubella
NH: National Health Service
PMT: Protection Motivation Theory
STI: Sexually transmitted infection
UK: United Kingdom
WHO: World Health Organization
